# FusionScan: accurate prediction of fusion genes from RNA-Seq data

**DOI:** 10.5808/GI.2019.17.3.e26

**Published:** 2019-07-23

**Authors:** Pora Kim, Ye Eun Jang, Sanghyuk Lee

**Affiliations:** 1 Ewha Research Center for Systems Biology (ERCSB), Ewha Womans University, Seoul 03760, Korea; 2 Department of Bio-Information Science, Ewha Womans University, Seoul 03760, Korea; 3 Department of Life Science, Ewha Womans University, Seoul 03760, Korea

**Keywords:** chromosomal translocation, fusion transcript, gene fusion, RNA-Seq, transcriptome sequencing

## Abstract

Identification of fusion gene is of prominent importance in cancer research field because of their potential as carcinogenic drivers. RNA sequencing (RNA-Seq) data have been the most useful source for identification of fusion transcripts. Although a number of algorithms have been developed thus far, most programs produce too many false-positives, thus making experimental confirmation almost impossible. We still lack a reliable program that achieves high precision with reasonable recall rate. Here, we present FusionScan, a highly optimized tool for predicting fusion transcripts from RNA-Seq data. We specifically search for split reads composed of intact exons at the fusion boundaries. Using 269 known fusion cases as the reference, we have implemented various mapping and filtering strategies to remove false-positives without discarding genuine fusions. In the performance test using three cell line datasets with validated fusion cases (NCI-H660, K562, and MCF-7), FusionScan outperformed other existing programs by a considerable margin, achieving the precision and recall rates of 60% and 79%, respectively. Simulation test also demonstrated that FusionScan recovered most of true positives without producing an overwhelming number of false-positives regardless of sequencing depth and read length. The computation time was comparable to other leading tools. We also provide several curative means to help users investigate the details of fusion candidates easily. We believe that FusionScan would be a reliable, efficient and convenient program for detecting fusion transcripts that meet the requirements in the clinical and experimental community. FusionScan is freely available at http://fusionscan.ewha.ac.kr/.

## Introduction

Fusion genes are important class of biomarkers in cancer studies. Numerous fusion genes have been established as cancer drivers including *BCR-ABL1* fusion in chronic myelogenous leukemia [1], *TMPRSS2-ERG* fusion in prostate cancer [2], *EML4-ALK* and *CD74-NRG1* fusions in non-small cell lung cancer [3,4], and *FGFR3-TACC3* in glioblastoma [5] and bladder cancer [6].

A number of algorithms and programs have been already published for fusion detection problem from RNA sequencing (RNA-Seq) data. Basic idea is to identify the split reads and discordant read pairs that map to two distinct genes. Subsequently, the exact fusion point is determined from the split reads where single mate reads overlap the fusion junction, with the fusion-encompassing reads used as supporting evidence. Early approaches following this scheme include FusionSeq [7], ChimeraScan [8], deFuse [9], FusionMap [10], TopHat-Fusion [11], and FusionHunter [12], as extensively reviewed by Wang et al. [13].

However, their performance varies dramatically in terms of precision, sensitivity (recall), and computational costs according to the mapping methods, filtering strategies, and parameter optimization. According to recent comparison where the performance of these tools was evaluated using synthetic and experimental datasets, no program showed satisfactory performance [14]. Programs with high sensitivity (ChimeraScan and TopHat-Fusion) predicted thousands of false-positives. Programs with low sensitivity (FusionMap, FusionHunter, and deFuse) still produced tens to hundreds of false-positives, unacceptable number for experimental confirmation, and had very limited overlap in the results.

Recent programs improved the performance by implementing diverse ideas. FusionQ used the concept of residual mapping to extend the short reads [15]. Similarly, BreakFusion combined the targeted assembly procedure to overcome the limits owing to short read length [16]. EricScript [17] improved the mapping accuracy by building exon junction reference and recalibration using BLAT [18]. Nevertheless, no programs achieved the accuracy over 50% of sensitivity and specificity simultaneously for the experimental datasets. Two programs are notable exceptions even though they have not been tested on public datasets. SOAPfuse used a library of fusion junction sequences by partial exhaustion algorithm and a series of filters to enhance confidence [19]. Analyzing two bladder cancer cell lines, they confirmed 15 cases out of 16 predictions, whereas deFuse identified 11 fusions of which 10 were confirmed by reverse transcriptase-polymerase chain reaction experiments. SOAPfusion implemented a novel masking and aligning procedure to achieve better sensitivity and false discovery rate than deFuse in the simulation test [20], but it needs further objective evaluation.

In this article, we report a novel algorithm FusionScan that implemented various strategies to enhance both the sensitivity and precision. We have compared the performance with other widely used programs using both experimental and simulated datasets. Our analysis demonstrated that careful mapping and extensive filtering processes were essential for good performance.

## Methods

### FusionScan algorithm

The goal of FusionScan is to identify fusion transcripts composed of combination of intact exons with high sensitivity and specificity. Thus, FusionScan requires multiple *split reads* that join intact exons of two different genes. This may miss cases where the fusion boundary exists inside the exon but the limitation is minor since most of important fusion markers are combination of intact exons thus far. This can be ascribed to the fact that the introns are much longer than exons in most eukaryotic genomes (e.g., ~27 times longer in the human genome). Furthermore, with the advances in sequencing throughput, the read length and sequencing depth of RNA-Seq has become long and deep enough to have multiple split reads including fusion boundaries in most cases.

**Fig. 1.**
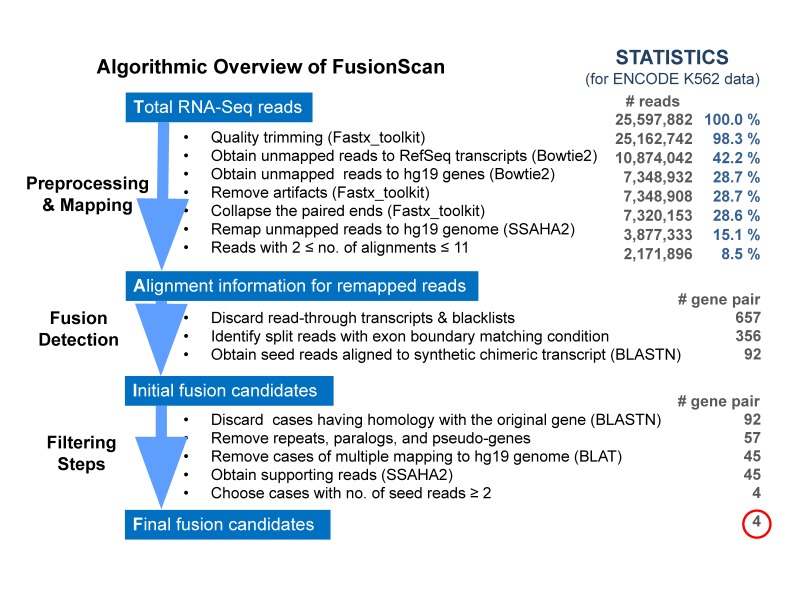
Overview of FusionScan algorithm. Computational pipeline is shown with programs used in each step. The statistics for processing K562 RNA sequencing data illustrates the effect of each procedure on reducing the candidates of split reads and fusion gene pairs. ENCODE, Encyclopedia of DNA Elements.

The algorithm consists of three main parts of preprocessing and mapping, fusion detection, and filtering steps as shown in Fig. 1. Usage of transcriptome model should be consistent through whole steps. We prefer the RefGene transcriptome model to the Ensembl because of its conservative criteria in modeling splice variants (e.g., total number of human transcripts for hg19 [GRCh37] assembly are 53,598 and 204,940 in the RefGene and Ensembl tracks of University of California Santa Cruz [UCSC] genome browser, respectively). Each step is optimized for reliable detection of fusion genes with high sensitivity and specificity as described below. To avoid confusion from naming, we will call two genes involved in the fusion as the head and tail genes according to the transcription direction of 5′→3′, and two exons adjacent to the fusion boundary as *fusion exons*.

### Preprocessing and mapping

A proper preprocessing to identify discordant reads and accurate alignment are the important starting points both for removing reads from normal transcripts for fast processing and for obtaining genuine split reads without loss. We find that these are the critical steps affecting the overall performance that have been overlooked in many cases.

(1) Quality trimming and artifact filtering were done by fastq_quality_trimmer (with the option of ‘-t 10 –l 38’ to keep reads with the minimum length > 38 bp of quality score > 10) and fastq_artifacts_filter in FASTX-Toolkit, respectively (http://hannonlab.cshl.edu/fastx_toolkit/).

(2) Mapping and removing regular reads were carried out in two step procedure. Bowtie2 v.2.1.0 [21] was used to map RNA-Seq reads to the human transcriptome of refGene from the UCSC genome annotation database for the hg19 (GRCh37). Unaligned reads were stored into a file with an option of ‘-un’ and they were realigned to the human genome, further removing reads mapped to the intronic or intergenic regions. Paired-end reads were processed independently in Bowtie mapping to identify discordant split reads. Then, the forward and reverse reads were joined and collapsed using fastx_collapser to produce unique unmapped reads only.

(3) Remapping unmapped reads was achieved by SSAHA2 alignment software [22]. We have tested several alignment tools for sensitive remapping including GMAP v.2013-11-27 [23], SSAHA2 v.2.5, Bowtie2 v.2.1.0, BWA v.0.7.5a [24], BLAT v.34, TopHat2 v.2.0.9 [25], and MapSplice v.2.1.7 [26]. We collected 269 fusion cases with known transcript sequence from TICdb [27] and ChimerDB 2.0 [28] (data available in the website). Then, we created the synthetic reads of variable length (50 bp, 76 bp, and 100 bp) with the fusion break point in the middle of the sequence, and remapped the synthetic read to the hg19 genome using various alignment tools. The number of fusion cases recovering correct alignments (i.e., identifying split reads successfully at the known fusion exons) was the highest in SSAHA2 by a narrow margin over Bowtie2 (Table 1). After extensive testing, we recommend using SSAHA2 with the option of ‘-solexa –skip 6 –cmatch 20 –best 5 –output pslx’ to set the seed length as 20 bp and to return five best alignments. Since this is the most time-consuming step, FusionScan supports the multi-thread option to split the unique fasta file and to run SSAHA2 alignment in parallel.

(4) Statistics of preprocessing and mapping are shown in Fig. 1 for the K562 RNA-Seq data of paired-end sequencing from the Encyclopedia of DNA Elements (ENCODE) project. Final number of remapped reads that may include the fusion candidates (2 ≤ no. of alignments ≤ 11) was reduced to 8.5% of the original data, thus speeding up down-stream analysis significantly.

### Fusion detection

FusionScan scans all read alignments looking for split reads whose aligned loci are apart by more than 50 kilo base pairs (kbp) in the genome, a condition that most known gene fusions satisfy. To ensure reliable alignment, we demand the minimum aligned length ≥ 20 bp on both sides and that the aligned parts cover more than 50% of the entire read. Two aligned loci of the split read should be contiguous within the range of ±10 bp over the transcript coordinate. Detailed conditions and explanations with illustrative figures are available at the online documentation.

Read-through transcripts are fairly common in the human transcriptome. Co-transcription and intergenic splicing (CoTIS) creates chimeric transcripts connecting exons of two neighboring genes [29]. Thus, we removed the read-through transcripts between two consecutive genes on the same strand with 5′ and 3′ ends accordant to the genome annotation. It should be noted that removing read-through cases may remove some genuine gene fusions arising from genomic deletions. We also support filtering by the blacklist of gene fusions at this stage. The black list may include unduly frequent gene fusions or fusion predictions that have failed in experimental validation.

Since FusionScan is designed to identify fusion genes specifically composed of intact exons from two participating genes, we apply two steps of examining fusion boundaries. First, we scan the fusion boundaries on the split read so that they appear within 6 bp from the intact boundaries of fusion exons (i.e., exon boundary offset ≤ 6 bp) to proceed to the next step. The candidate split read is then realigned to the synthetic sequence of combined fusion exons using the bl2seq tool of BLAST with the word size of 20 [30]. Again, the minimum aligned length is 20 bp on both sides with the minimum percent identity ≥ 95%. Split reads satisfying all conditions given above are the seed reads that would strongly support the fusion event. For K562 cell line data shown in Fig. 1, the exon boundary condition and realignment against the synthetic chimeric transcript reduced the number of candidates considerably, and we obtained 92 fusion gene pairs for the filtering procedure.

### Filtering steps

Since most programs for fusion gene prediction yield too many false-positives, extensive filtering is essential for reliable performance. In an effort to enhance the precision of the prediction (i.e., small number of false-positives), we have implemented several filtering strategies to prevent accidental alignment leading to false split reads as follows:

(1) Homology filter was applied if the nucleotides of 14 bp length before and after the fusion point were homologous to the original sequences of two participating genes. Bl2seq was used to detect homology with the word size of 10.

(2) Filters for repeat regions, paralogs, and pseudo-genes were implemented as well. We discarded the seed reads that were aligned within the repeat regions obtained from the Repeat-Masker [31] track in the UCSC genome browser. Similarly, gene fusions with paralogous genes obtained from the Duplicated Genes Database [32] or pseudo-genes obtained from the Human Genome Organisation (HUGO) database [33] were removed from the candidates.

(3) In spite of extensive filtering as described above, we still observed many cases where the split read had alternative alignment of similar or better quality elsewhere in the genome. We implemented the multiple mapping filter by running the local version of BLAT v.34 (using the same option as the web version of BLAT) for seed reads to identify such cases of ambiguous multiple mapping (sequence identity > 95%) and removed those from the fusion candidates.

(4) Finally, we choose the fusion candidates with multiple seed reads as reliable (i.e. the minimum number of seed reads = 2).

For K562 cell line data, FusionScan predicted 4 fusion gene pairs in total, and 3 of those were validated experimentally. The workflow in Fig. 1 shows that (1) the homology filter was not effective for this data, (2) removing repeats, paralogs, and pseudo-genes is an important step of reducing 35 candidates, (3) recalibration with BLAT alignment is helpful to reduce 12 additional candidates. However, the condition of multiple seed reads was most critical to yield only 4 fusion candidates.

### Curative tools

Even after using various elaborate filters described above, it is often necessary for users to examine the alignment explicitly. We have developed several tools to facilitate visual inspection by users.

**Fig. 2.**
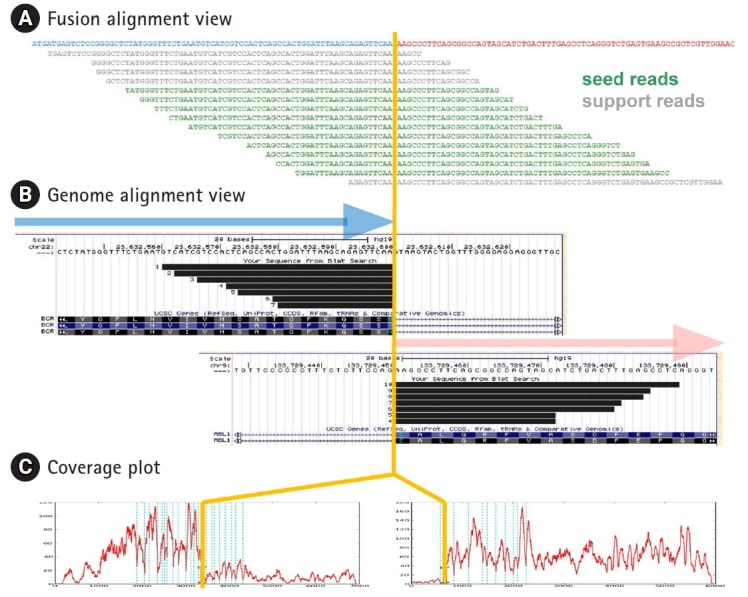
Alignment and coverage plots. *BCR-ABL1* gene fusion detected from RNA sequencing data of K562 cell line is shown as an example. (A) Fusion alignment view is the read alignment of seed and support reads on hypothetical fusion transcript. (B) Genome alignment view shows the alignment of split reads on the University of California Santa Cruz (UCSC) genome browser for head and tail genes obtained by BLAT alignment tool. (C) Coverage plots on transcript coordinate show abrupt change in read depth at the fusion boundary for both head and tail genes. Blue vertical lines indicate the exon boundaries in each gene.

(1) Alignment plot is of great help to verify the genuine fusion events. We provide two different types of alignment plot as shown in Fig. 2. Fusion alignment view shows the alignment of fusion reads onto the synthetic fusion sequence. Progressive tiling pattern is the most desirable feature for the genuine fusion genes. Genome alignment view shows the alignment of fusion transcripts separately for head and tail genes as a custom track in the UCSC genome browser (Fig. 2B).

(2) Coverage plot from next-generation sequencing data provides valuable information on genomic or transcriptome structures. For example, abrupt depth change at exon boundaries often indicates the gene fusion or alternative splicing events. FusionScan provides coverage plots for head and tail genes. As shown in Fig. 2C, both the head and tail genes showed abrupt jump at the fusion boundaries in accordance with the BCR-ABL1 fusion event. Testing many RNA-Seq data, we found that this abrupt change applied well for most well-known cases, especially for the tail kinase genes.

(3) Split seed reads are the most direct evidence of gene fusion. In FusionScan, we acknowledge the split reads as the seed only if both sides were aligned to fusion exons over 20 bp long. In cases where only one side met the condition and the other side had a shorter aligned part, we classify them as the *support reads*, which still serve as indirect but good evidence of fusion event. To identify support reads, we realign all RNA-Seq reads to the synthetic chimeric transcripts using SSAHA2 again, and the result is reported with the number of seed reads or used in the fusion alignment plot. This process is optional since it demands realignment of all RNA-Seq reads, taking significant amount of computation. The fusion alignment view may include the support reads as shown in Fig. 2A.

## Results

Since a number of fusion detection programs are already available in public, it is critical to compare the performance of programs objectively. We have carried out the performance evaluation tests for FusionScan, SOAPfuse v.1.26, deFuse v.0.6.1, FusionHunter v.1.4, FusionMap v.2012-08-12, and TopHat-Fusion v.2.0.9 using both experimental and simulation data sets. All programs were run with the default options using the recommended mapping programs and transcriptome model as summarized at the bottom of Table 2. For TopHat-Fusion, we used the output from the TopHat-Fusion-Post that reduced the false-positives using BLAST search since it produced too many false-positives without the -Post option. The feature of blacklist was not used for fair comparison.

### Comparison of fusion discovery tools using experimental data from three cancer cell lines

#### The data

NCI-H660 is a prostate cancer cell line where two fusion genes (TMPRSS2-ERG and EEF2-SLC25A42) have been verified to play important roles in tumorigenesis. We downloaded the RNA-Seq data from the FusionSeq website [7], which included 6.5 million paired-end reads of 51 bp long.

K562 cell line has long been the standard of leukemia studies where the most famous BCR-ABL1 fusion was identified. Single-end RNA-Seq data for long polyA cytosol mRNAs was downloaded from the Caltech RNA-seq group at the UCSC ENCODE web site. The data includes 12.8 million reads of paired-end sequencing with 76 bp read length. Three cases of gene fusion were known for the K562 cell line [34].

One of the most extensively studied samples for gene fusion is the MCF-7 breast cancer cell line. The Caltech RNA-Seq group includes RNA-Seq data of MCF-7 cell line as well (SRR521521 in SRA database). The data contains 40 million reads of paired-end sequencing with 76 bp read length. Sakarya et al. [35] independently studied gene fusions in the MCF-7 cell line using 80 million reads produced by SOLiD paired-end sequencing. They validated 23 gene fusions using TaqMan fusion assays, which were used as gold standards for our benchmark test.

Three data sets of cancer cell lines from public resources represent diverse situations such as different cell types, sequencing depth, single and paired reads, and different read lengths, thus being expected to provide objective result in the comparison test.

#### Performance comparison

The result from six programs for fusion detection based on RNA-Seq data is summarized in Table 2. For fair comparison, we filtered out all cases with the number of seed reads = 1 since FusionScan required the number of seed reads ≥ 2. This may remove some true positives in other programs, but certainly helps in removing false-positives. We calculated the precision and recall rates since the true-negatives are difficult to prove in gene fusion discovery. It should be noted that we did not penalize other programs for giving wrong direction (i.e., reversed head and tail genes).

In general, the precision and recall rates are contradictory to each other. FusionScan achieved the best in the precision rate (60%) and in the overall performance measured by F1 score, the harmonic mean of precision and recall rates. SOAPfuse was the best in the recall rate (93%) but its precision rate was just 20%, producing lots of false-positives. Fusion-Hunter achieved the precision rate of 46% by sacrificing the recall rate to 39%, missing too many true positives. TopHat-Fusion showed fairly good performance mainly because of recent implementation of extensive filtering scheme in the TopHat-Fusion-Post option.

For experimental biologists or clinicians who carry out validation experiments with limited amount of samples, the precision rate is the most critical attribute. Thus, it is important to note that FusionScan achieved the precision rate of 60% without losing the recall rate considerably (79%). The difference with other programs is substantial, including FusionHunter that achieved excellent performance in recent comparison test by the SOAPfusion study [20]. It should be noted that one fusion case of C16orf45-ABCC1 was not predicted by all programs, which may suggest that fusion reads for this case were not present in the Caltech RNA-Seq data unlike the SOLiD sequencing data by Sakarya et al. [35]. Excluding this case, the recall rate of FusionScan increases to 81.5%.

**Fig. 3.**
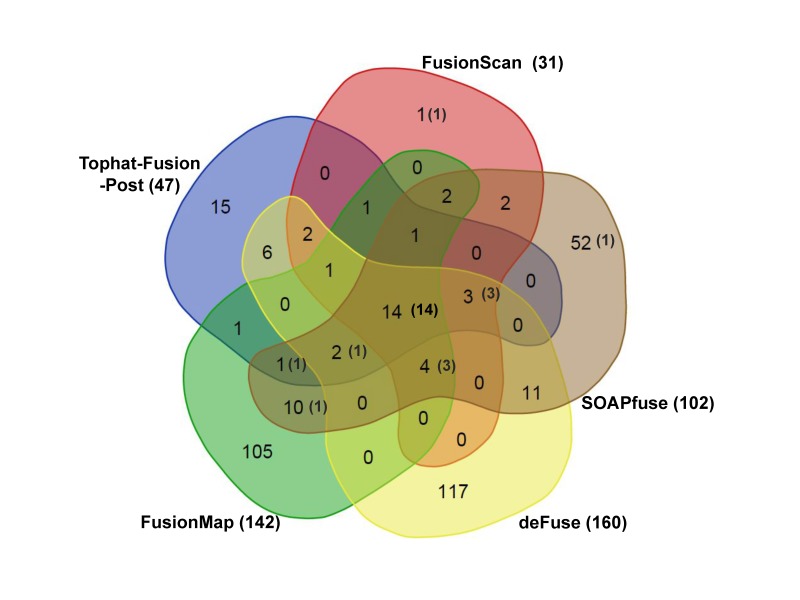
Venn diagram of fusion predictions. We show the total number of predicted fusion genes for all three cell lines from five different programs. Numbers in the parenthesis indicate the number of true positive cases.

The prediction results from five tools are further illustrated as a Venn diagram in Fig. 3, excluding FusionHunter that missed many true positives. Common hits would have better chance to be genuine fusion cases. FusionScan showed the most common hits from more than three programs (28 out of 31 cases). Importantly, FusionScan had only one singleton prediction, which strongly supports the reliability FusionScan’s predictions. FusionMap, deFuse, and SOAPfuse had a number of singleton predictions, most of those being expected to be false-positives.

### Comparison of fusion discovery tools using simulation data sets

Testing with experimental datasets is objective and reliable since it reflects diverse situations and experimental conditions that could not be mimicked in simulation studies. However, the scope of benchmark test is limited with small number of known fusion cases and with experimental settings under specific conditions. Thus, we carried out the benchmark test using simulation datasets as well to estimate the performance of each program in different conditions such as variable read length and coverage.

#### Preparing the simulation data

Positive cases of fusion gene were artificially constructed by joining two exons of randomly chosen genes, isoforms, and exons in the given order. Adjacent genes were avoided in the selection to exclude read-through transcripts. Using the transcriptome model of refGene, we have generated 10,000 fusion cases to sample diverse sequence characteristics for the benchmark test.

For each fusion case, we prepared a synthetic fusion transcript by concatenating the 5′ side of the head transcript and 3′ side of the tail transcript at the fusion boundary. Random nucleotide position was selected to make a paired end read of desired read lengths (50 bp, 75 bp, or 100 bp) until the pre-determined depth of 10×, 30×, or 50× was achieved. We also demanded the minimum coverage of transcript of 95% (i.e., less than 5% of nucleotides not covered by a sequencing read). The insert size of the paired end reads were selected randomly following the normal distribution with the average insert size of 100 bp and with standard deviation of 10 bp.

Compared to the existing simulation methods that usually add hundreds of synthetic fusion transcripts to the transcriptome model (e.g., RefGene or Ensembl) and run a simulator for producing paired-end sequencing data [19,20], our procedure of preparing simulation datasets has the advantage of reflecting diverse characters of fusion gene sequences. Weakness of not taking imbalanced sequencing depth or sequencing errors into account can be alleviated by the comparison test using real datasets as previously described. The list of 10,000 fusion cases and simulated paired-end sequencing data are available at the website.

#### Performance comparison

**Fig. 4.**
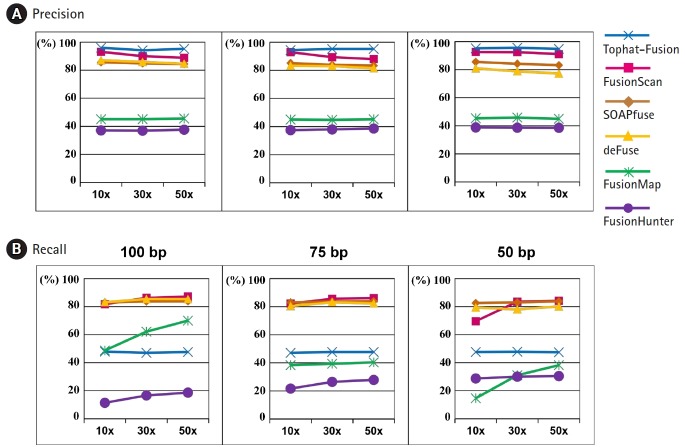
Precision and recall curves for performance evaluation using simulation data sets. (A) Precision rates at the read length of 100 bp, 75 bp, and 50 bp. (B) Recall rates at the read length of 100 bp, 75 bp, 50 bp. 10×, 30×, and 50× indicate the sequencing depth of the simulation data.

The precision and recall curves from six different programs for fusion detection are shown in Fig. 4 for various sequencing depths and read lengths. Here, we used the default settings of each program for the minimum number of seed reads, instead of demanding two seed reads at least as for the experimental datasets.

TopHat-Fusion-Post showed the highest precision rate consistently but its recall rate was close to 50%. FusionScan was the second to TopHat-Fusion in the precision and the best in the recall rate. At the read length of 100 bp and 50× depth, a common practice with recent advances in sequencing technology, FusionScan showed the precision and recall rates of 89% and 87%, respectively. The performance of SOAPfuse and deFuse was slightly inferior to FusionScan in precision and was comparable in the recall rates.

As the sequencing depth increased, the recall rates were improved in all programs. FusionMap and FusionHunter showed substantial variation. The precision rates, however, were fairly independent of sequencing depth and read length.

Overall, the simulated test showed that three programs (FusionScan, SOAPfuse, and deFuse) achieved comparable performance with a slight advantage to FusionScan in the precision. TopHat-Fusion’s prediction is reliable, but it misses many true positives as well.

### Implementation and computational resources

FusionScan algorithm was developed using Java (JDK1.7) and Python languages. It further requires many third-party programs such as Bowtie2, SSAHA2, BLAT, bl2seq, samtools and FASTX-Toolkit. Thus, it is highly recommended to run FusionScan in Linux environment with the Java Runtime Environment 1.7 or later.

**Fig. 5.**
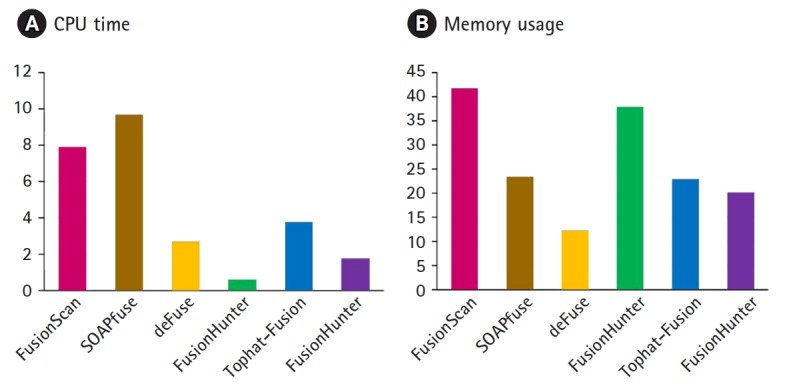
Comparison of CPU time (A) and memory usage (B). CPU time and memory usage are shown in hours and GB, respectively. RNA sequencing data for K562 cell line from the Encyclopedia of DNA Elements (ENCODE) project (SRR521464) was analyzed on a 64-bit machine AMD Opteron Processor 6176 (2.3 GHz, 8 core) with 32 GB RAM.

We measured the CPU time and memory usage the K562 RNA-Seq data are compared in Fig. 5. FusionScan and SOAPfuse took the longest CPU time mainly to achieve the high recall rates. For example, quality trimming with the option of ‘-t 20 –l 40’ instead of ‘-t 10 –l 38’ decreased the run time by half in FusionScan, but lost a few true positives in benchmark testing with 3 cell line datasets. Measuring the CPU time spent for each step of workflow, the preprocessing and mapping took almost half of the total CPU time.

## Discussion

For both the real and simulated datasets, the results show that FusionScan provides reliable predictions in fusion discovery under different sequencing coverage and read length. Even though the general trends were similar between the two datasets, the precision was much worse for the experimental datasets. This indicates that there exist many factors influencing the prediction accuracy in reality (e.g., coverage imbalance, sequencing errors, and sequence polymorphisms). Thus, the result from simulation data should be taken cautiously. Interestingly, SOAPfuse achieved better recall rate for the experimental datasets.

FusionScan was the only program with the precision rate over 50%. The enhanced performance of FusionScan may be ascribed to several points as follows.

(1) Accurate read alignment is absolutely critical. We have selected SSAHA2 as the most sensitive mapping program through a test with known fusion transcripts. This process minimizes the loss of true positives from the start. In a similar effort, SOAPfusion used a special aligner that masked the intronic regions from the transcripts. It should be noted that other alignment tools in Table 1 may do better with extensive adjustment of optional parameters. We provide the full set of known fusion cases for testing such possibility.

(2) Reads with alternative mapping positions should be analyzed cautiously. Many false-positives from other programs had their seed reads mapped to other positions concordantly with similar mapping quality. FusionScan removed those ambiguously mapped reads using extensive filters afterwards. Critical two steps were removing repeats, paralogs, and pseudo-genes and recalibration with BLAT alignment as shown in Fig. 1.

Predicting fusion genes from RNA-Seq data is a procedure full of optimization steps. For example, we have noticed that four true positive cases in MCF-7 cell line were filtered out in FusionScan at the final step since they had only one seed read. Relieving the minimum number of seed reads as 1 or using support reads as the basis of rescuing those cases would introduce too many false-positives.

We used the preliminary version of FusionScan and TopHat-Fusion to build the ChimerDB 3.0 update [36]. Fusion gene candidates were obtained by analyzing RNA-Seq data from The Cancer Genome Atlas (TCGA). Of note, STAR-Fusion was recently released in bioRxiv and GitHub. It achieved comparable performance to FusionScan in terms of precision and recall rate. JAFFA is another latest pipeline for fusion detection that utilizes read assembly into transcripts before fusion detection [37]. This assembly-based method certainly achieved a good performance in favorable conditions with large number of high quality reads. But its performance decreased rapidly in bad conditions where misassembles led to many false-positives and negatives. A latest benchmark test was carried out to evaluate the performance of 12 popular fusion detection tools and provided some guidelines even though their test datasets are rather limited [38].

In conclusion, FusionScan made a reasonable compromise between precision and recall rates, achieving 60% and 79%, respectively, in tests using experimental datasets. With implementation of several curative tools facilitating validation of fusion transcripts, we believe that FusionScan would be a reliable tool for detecting fusion transcripts, meeting the conservative conditions required for clinical and experimental studies.

## Figures and Tables

**Table 1.** Comparison of RNA-Seq alignment programs

Mapping program	No. of correct alignments out of 269 known fusion transcripts^a^		
	50 bp	75 bp	100 bp
GMAP	59	28	3
SSAHA2	237	248	252
Bowtie2	242	245	248
BWA	1	238	244
BLAT	218	225	226
TopHat2	227	228	226

All alignment tools were run with default options. RNA-Seq, RNA sequencing.

a Two hundred sixty-nine known fusion transcripts were collected from TICdb and ChimerDB 2.0.

**Table 2.** Identification of known fusion genes by various fusion detection tools

Sample	Known (Gold) fusion genes	FS	SF	dF	FH	FM	THF
NCI-H660 (2)	TMPRSS2-ERG	●	●	●	●	●	●
	EEF2-SLC25A42	●	●	●	●	-	●
	TP/FP	2/0	2/16	2/11	2/1	1/1	2/1
	Precision	1.0	0.11	0.15	0.67	0.50	0.67
	Recall	1.0	1.0	1.0	1.0	0.50	1.0
K562 (3)	BCR-ABL1	●	●	●	-	●	●
	NUP214-XKR3	●	●	○	●	●	●
	BAT3-SLC44A4	●	●	○	-	●	●
	TP/FP	3/1	3/7	3/27	1/1	3/12	3/0
	Precision	0.75	0.30	0.10	1.0	0.20	1.0
	Recall	1.0	1.0	1.0	0.33	1.0	1.0
MCF-7 (23)	USP31-CRYL1	●	●	○	○	○	●
	ARFGEF2-SULF2	●	●	○	-	○	●
	TXLNG-SYAP1	●	●	●	-	○	●
	DEPDC1B-ELOVL7	●	●	●	○	●	●
	SYTL2-PICALM	●	●	●	○	●	●
	RPS6KB1-DIAPH3	-	●	●	-	-	-
	AHCYL1-RAD51C	-	●	-	●	●	●
	TAF4-BRIP1	-	●	-	-	○	-
	POP1-MATN2	●	●	●	-	○	-
	GCN1L1-MSI1	●	●	●	-	-	●
	ESR1-CCDC170	●	●	●	●	○	●
	SMARCA4-CARM1	●	●	●	-	○	●
	MYO6-SENP6	●	●	●	●	○	●
	ADAMTS19-SLC27A6	●	●	●	●	○	●
	GATAD2B-NUP210L	-	●	●	-	○	●
	SLC25A24-NBPF6	●	●	●	-	●	-
	ATXN7L3-FAM171A2	●	●	●	-	●	-
	C16orf62-IQCK	●	●	●	-	●	●
	TBL1XR1-RGS17	●	-		-	-	-
	BCAS4-BCAS3	●	●	●	●	-	●
	RPS6KB1-TMEM49	●	●	●	-	○	●
	ABCA5-PPP4R1L	-	●	-	-	-	**-**
	C16orf45-ABCC1	-	-	-	-	-	-
	TP/FP	17/14	21/83	18/132	8/11	17/126	15/26
	Precision	0.55	0.18	0.12	0.42	0.12	0.37
	Recall	0.74	0.91	0.78	0.35	0.74	0.65
Overall	Precision	0.60	0.20	0.12	0.46	0.13	0.43
	Recall	0.79	0.93	0.82	0.39	0.75	0.71
	F_1_ score	0.68	0.33	0.21	0.42	0.22	0.53
Mapping program		SSAHA2	SOAP2	GMAP	Bowtie	GSNAP	Bowtie
			BWA				
Transcriptome		RefGene	Ensembl	Ensembl	RefGene	RefGene	Ensembl

‘●’ and ‘○’ indicate that the case was predicted successfully, with direction reversed in ‘○’. Precision = TP/(TP + FP), Recall = TP/(TP + FN), F_1_ score = 2 × precision×recall/(precision + recall). FS, FusionScan; SF, SOAPfuse; dF, defuse; FH, FusionHunter; FM, FusionMap; THF, TopHat-Fusion; TP, true-positive; FP, false-positive; FN, false-negative.
